# A systematic strategy for the investigation of vaccines and drugs targeting bacteria

**DOI:** 10.1016/j.csbj.2020.06.008

**Published:** 2020-06-12

**Authors:** Fangfang Yan, Feng Gao

**Affiliations:** a Department of Physics, School of Science, Tianjin University, Tianjin 300072, China; b Frontiers Science Center for Synthetic Biology and Key Laboratory of Systems Bioengineering (Ministry of Education), Tianjin University, Tianjin 300072, China; c SynBio Research Platform, Collaborative Innovation Center of Chemical Science and Engineering (Tianjin), Tianjin 300072, China

**Keywords:** Subtractive genome analysis, Molecular dynamics simulation, Target analysis, Infectious and epidemic diseases, Bacteria

## Abstract

Infectious and epidemic diseases induced by bacteria have historically caused great distress to people, and have even resulted in a large number of deaths worldwide. At present, many researchers are working on the discovery of viable drug and vaccine targets for bacteria through multiple methods, including the analyses of comparative subtractive genome, core genome, replication-related proteins, transcriptomics and riboswitches, which plays a significant part in the treatment of infectious and pandemic diseases. The 3D structures of the desired target proteins, drugs and epitopes can be predicted and modeled through target analysis. Meanwhile, molecular dynamics (MD) analysis of the constructed drug/epitope-protein complexes is an important standard for testing the suitability of these screened drugs and vaccines. Currently, target discovery, target analysis and MD analysis are integrated into a systematic set of drug and vaccine analysis strategy for bacteria. We hope that this comprehensive strategy will help in the design of high-performance vaccines and drugs.

## Graphical abstract

**Figure:**
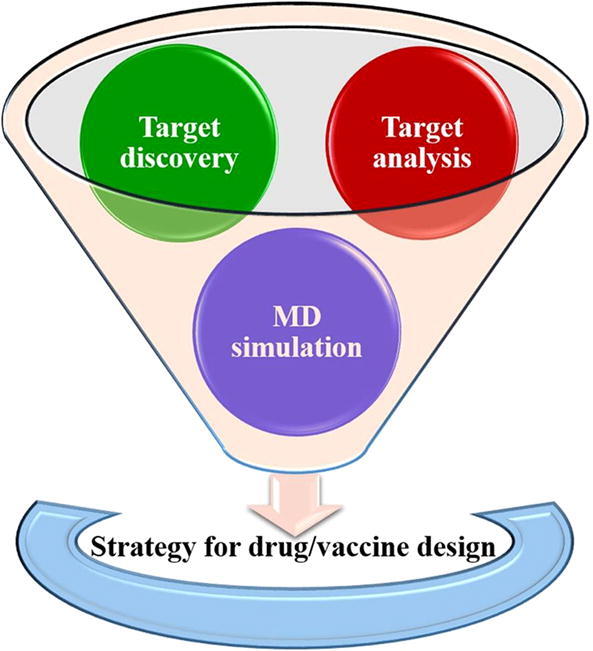

## 1 Introduction

Human health is continuously threatened by various infectious diseases and large-scale epidemics caused by bacteria, medicines and vaccines are important means for the treatment of human diseases. In the past, owing to the fact that technology and resources still had not matured sufficiently, effective drugs or vaccines could not be developed promptly to cure the diseases under pressing epidemic situations and therefore epidemics or infectious diseases always cause a panic. With developments in medicine and technology, several vaccines and drugs were gradually developed. However, a few of them failed to achieve the desired effect, or potentially interfered with other normal functions and produced certain adverse effects [1]. Even presently, the development of innovative drugs still poses great challenges, such as extreme complexities, high risk, long development cycle and huge investment [2], [3], [4]. Thus, ensuring rapid, safe and effective development of drugs and vaccines has always been an urgent problem. The development of vaccines and drugs can be roughly divided into preclinical and clinical development, in which preclinical development plays a dominant role in the whole process [5]. If a candidate vaccine/drug is not proven to be safe and effective in preclinical studies, no further clinical studies are required. In preclinical studies, drug discovery is the first step in the drug development, which aims to achieve breakthrough progress. Therefore, we pay extra attention to the discovery of new drugs and vaccines in this review.

Investigation of new drugs and vaccines has continued throughout the history of human development. Initially, researchers isolated and identified the effective components to treat various diseases mainly from natural products [6]. However, employing natural products has certain challenges in practical applications, such as their low solubility and stability. Therefore, it is necessary to structurally modify the effective natural components. In 1796, Edward Jenner was successful in preventing a smallpox virus infection using a vaccinia vaccine. This achievement was the first victory in the history of vaccine development, and the beginning of vaccinology and immunology. Unfortunately, no new vaccine has emerged in the more than 100 years since the discovery of the first. At the end of the 19th century, Louis Pasteur et al. developed the anthrax vaccine and proposed the principle of vaccinology [7], which was a big step in the study of vaccines, and led to the development of a variety of vaccines to resist the corresponding pathogens [8], [9], [10]. Until 1932, the structural modification of drug molecules was first guided by a theory proposed by Erlenmeyer, which opened the way for further development in drug theories [11]. Subsequently, a quantitative structure-activity relationship (QSAR) was developed by Hansch et al. in 1964 [12]. QSAR can improve the success rate of candidate drugs in clinical experience, and lays a theoretical and practical foundation for quantitative drug design. Simultaneously, the development of bacterial vaccines had also progressed further before the mid-20th century. Since the late 20th century, bioinformatics, molecular biology, pharmacy, immunology, microbiology, and other related disciplines have developed rapidly, which has allowed new opportunities in the development of bacterial vaccines and drugs. Techniques for proteomic and genomic analyses have been further developed, and a large number of proteins and their coding genes have been discovered. At present, the designing of proteome- or genome-based bacterial drugs and vaccines has emerged as the new direction [13].

According to the published literature [14], [15], [16], the genome/proteome-based drug and vaccine design mainly involves four steps: selection and identification of drug target, optimization of the target molecules, discovery of compounds and peptide epitopes, and optimization of the compounds and peptide epitopes. The generation and availability of a large amount of genomic data have enabled the identification of effective targets through computational genomics methods, and completely changed the threat of pathogens to humans [17]. Among these genomics methods, the comparative subtractive genome approach has laid the foundation for target discovery and become an extensive tool for mining promising therapeutic targets [18], [19]. Other methods, including core genome [20], replication-related proteins [21], transcriptomics [22] and riboswitches analyses [23] have also garnered increasing attention for exploration of drug targets. Furthermore, target prioritization is an indispensable step in the design of drugs and vaccines. A three-dimensional (3D) model [24] for the target proteins, epitopes and drugs can be successfully predicted and constructed based on an in-depth analysis of the drug/vaccine targets. In addition, MD analysis of these modeled drug/epitope-protein complexes is a necessary standard for testing the effectiveness of drugs and vaccines. By MD simulation, the binding ability of inhibitors/peptides to proteins and the conformational changes of target proteins will be well reflected [25], [26].

Therefore, this review focuses on a combination of three important sections (target discovery, target analysis and MD analysis) to discover the preclinical inhibitors and vaccines that target bacteria-related diseases. First, we introduce five universal methods for exploring the targets: comparative subtractive genome, core genome, replication-related proteins, transcriptomics, and riboswitches analyses. Then, we summarize the basic process of the drug and vaccine design, which mainly includes target optimization, screening of drugs and vaccines, and optimization of drugs and vaccines. Finally, MD simulation and some advanced methods based on MD trajectory are described in detail.

## 2 Target discovery

**Fig. 1:**
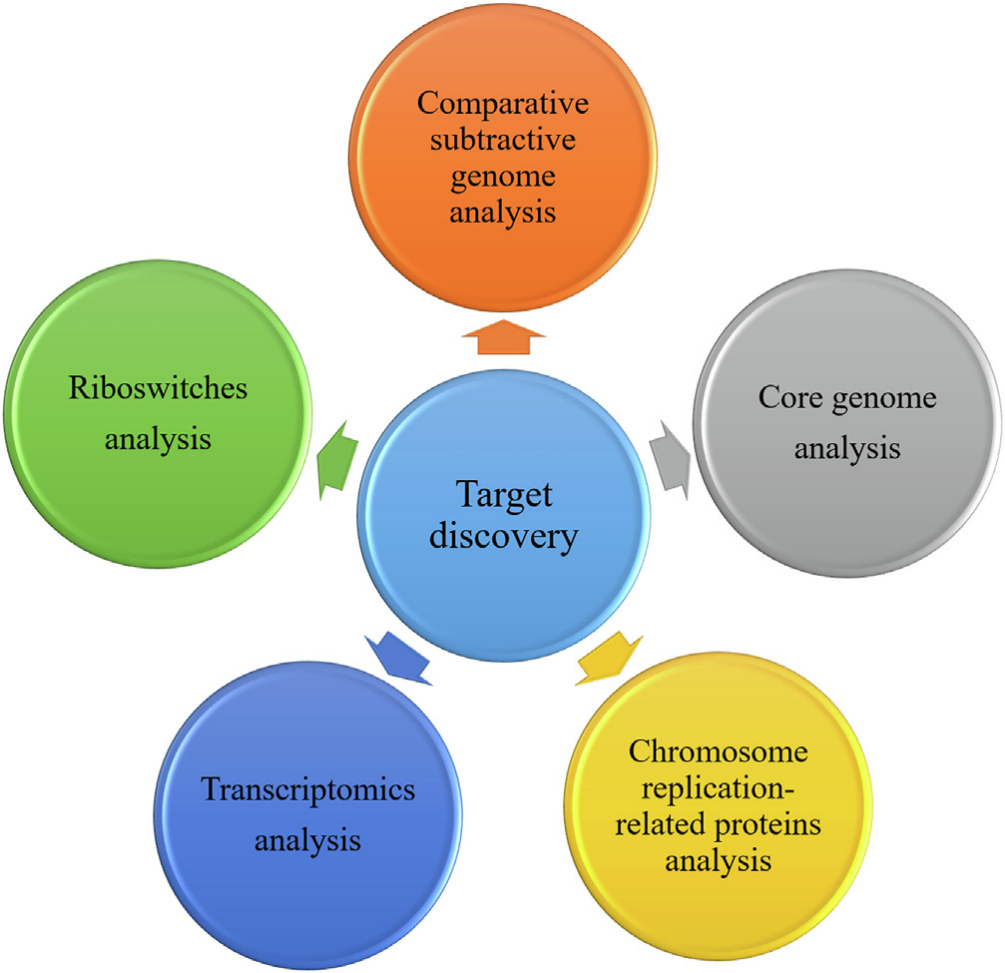
Multiple ways for drug discovery.

Exploring the therapeutic targets in bacteria is the first and crucial step in developing efficient vaccines and drugs. Certain essential proteins and proteins involved in basic cellular processes can serve as potential targets for novel antimicrobial agents. In this section, we summarize five analytical methods for exploration of drug targets (Fig. 1).

### 2.1 Comparative subtractive genome analysis

**Fig. 2:**
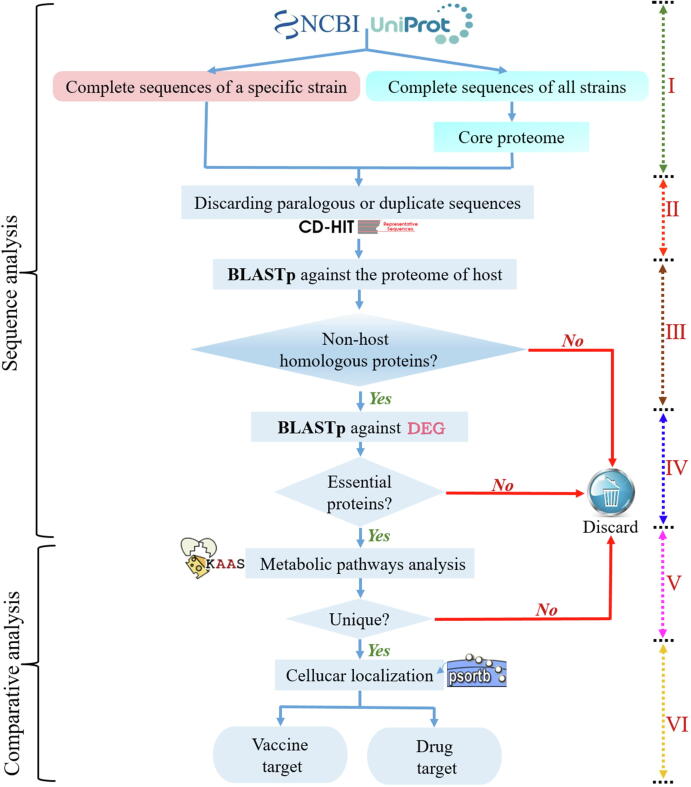
Workflow for screening potential drug and vaccine targets from complete sequences using comparative subtractive genome analysis and core genome analysis, respectively.

For the actual target selection, the potential candidate targets should be necessary for bacterial growth and reproduction, non-homologous to the host proteins, and have a unique metabolic pathway different from the host. With the aim of finding essential and non-homologous targets with unique metabolic pathways, subtractive genome analysis is selected to analyze the bacterial proteome through layers of screening. Since Sakharkar et al. first proposed the subtractive genome approach [1], many researchers have used this method to analyze drug and vaccine targets, which has immense potential for future experimental design of novel drugs and vaccines. For example, Sharma et al. revealed the target candidates for *Lymphatic filariasis*in in 2016 [18], and Sudha et al. investigated the drug targets and vaccine candidates for *Clostridium botulinum* in 2019 [19] using this method. In the following sections, we summarize the target screening process using the subtractive genome method. The detailed and complete workflow is shown in Fig. 2.

#### 2.1.1 Getting the complete sequences of bacteria

According to published studies [19], [27], the complete sequences of bacteria for subtractive genome analysis are mainly retrieved as files in FASTA format from the National Center for Biotechnology Information (NCBI) [28] and Universal Protein (UniProt) [29] databases (Fig. 2I), which are the most informative and extensive protein databases.

#### 2.1.2 Removing paralogous or duplicate sequences

The rapid emergence of next generation sequencing (NGS) technology has led to an explosive growth in biological sequence data, and the removal of redundant or duplicate sequence data has become one of the significant challenges to subsequent bioinformatics analyses [30]. Luckily, Li et al. created a fast online program CD-HIT [31] to search representative protein sequences based on the possible correlation and homology of certain sequences (Fig. 2II), alleviating the problem of calculation and analysis to some extent. To date, CD-HIT has been widely used to discard redundant or duplicate sequences by comparing the similarities between two sequences with expected threshold values.

#### 2.1.3 Eliminating host-homologous sequences

Eliminating sequences that are homologous to the host, is a crucial operation in this process. If the target protein is homologous to the one in the host, the designed drug may produce nonspecific interactions with the host protein, resulting in certain negative effects [32]. Therefore, selecting proteins that are non-homologous to those in the host is necessary. Basic local alignment search tool (BLAST) [33] is the best choice for this requirement. In this section, BLASTp is applied by numerous researchers to perform a similarity search by comparing non-paralogous proteins with the entire host proteome (Fig. 2III), with the expectation value (e-value) set to widely used threshold 0.0001 [14], [34], [35]. Finally, the sequences that are homologous to those in the host are deleted.

#### 2.1.4 Screening the essential proteins in bacteria

Choosing the essential proteins in bacteria, is another crucial step in this process. The essential proteins in the bacterial proteome are crucial for maintaining their life activities under specific conditions and vital importance for their survival, and any blocking of their functions will lead to cell death [36]. Hence, inhibiting the activity of such essential proteins can greatly improve the therapeutic effect in bacterial diseases. To select the essential proteins in bacterial proteome, an essentiality analysis is conducted on the non-homologous proteins. In subtractive genome analysis, it is common for users to perform a BLAST search against the Database of Essential Genes (DEG) [37] to remove non-essential proteins (Fig. 2IV) [38], [39], [40]. Since the DEG database was developed by Zhang et al. in 2004 [37], the content of this database has been updated continually and a large number of essential genes in prokaryotes and eukaryotes have been included [41]. Collection of a larger amount of essential gene data and availability of flexible BLAST tools [42] would contribute even more to the prediction of essential genes or proteins.

#### 2.1.5 Metabolic pathway analysis

A metabolic pathway analysis [43] is performed on the non-homologous essential proteins by utilizing the Kyoto Encyclopedia of Genes and Genomes (KEGG) [44] Automatic Annotation Server (KAAS) [45] to identify the metabolic pathway of the targets, and similarity searches with BLASTp are conducted for all existing proteins against the latest KEGG database (Fig. 2V). Meanwhile, the metabolic pathways of the bacteria and their hosts also need to be compared. If the protein is involved in a unique metabolic pathway, it is marked for subsequent analyses; otherwise, the protein is removed from the proteome under consideration. Through this comparative pathway method, the non-homologous essential proteins following unique metabolic pathways can be mapped, and these proteins can be key targets for the treatment of diseases.

#### 2.1.6 Subcellular localization analysis

Predicting the subcellular localization of bacterial proteins is critical to the identification of target proteins, and can quickly provide information about the protein function [46], [47]. An ideal candidate protein for a vaccine should interact with the extracellular environment and trigger the immune system of the host effectively; therefore, proteins distributed on the extracellular and outer membranes are considered effective vaccine candidates [48]. Meanwhile, it has been demonstrated that cytoplasm-related proteins can be effective drug targets [49]. At this stage, the remaining therapeutic targets are subjected to subcellular localization analysis to identify potential drug and vaccine candidates by using the most accurate and user-friendly PSORTb server [50] (Fig. 2VI). Besides, some other verified methods (CELLO [51], PA-SUB [52], SignalIP [53], Phobius [54] and ngLOC [55]) can be combined with PSORTb to achieve a more precise prediction of subcellular localization for predicted targets.

After subtractive genome analysis, the putative drug and vaccine targets have been identified separately, which is the cornerstone of future drug and vaccine design.

### 2.2 Core genome analysis

Studies have confirmed that the bacterial core genome plays an important role in their growth, and is also related to the essence of the species [56], [57]. The core genome dataset comprises the common genes in all the available strains of species, and the genes that belong to the core genome are closely related to the nature of the species [58], which makes core genome analysis a reasonable method to address the difficulty in obtaining therapeutic targets. Therefore, comparative subtractive genome analysis based on the core genome of bacteria is another method used to detect targets. In contrast to the subtractive genome method based on essential genes, the first step in core genome analysis is obtaining the complete sequences of all strains for a particular species (Fig. 2). According to recently published works [59], [60], [61], the core genome can be probed by Pan-Genome Analysis Pipeline (PGAP) [62], EDGAR tool version 2.0 [63], etc.

### 2.3 Chromosome replication-related proteins analysis

Chromosome replication-related proteins can also be used as potential targets for exploring novel and effective antimicrobials. It is well known that all bacterial cells undergo chromosome replication before they can be split into two identical daughter cells. Chromosome replication-related proteins are essential for maintaining cellular activity and the replication process of chromosomes, and represent a promising target class [21], [64]. Unfortunately, other than nonsteroidal anti-inflammatory drugs [65] and aminocoumarin [66], there are few available antimicrobials for targeting the bacterial chromosome replication. Therefore, identifying potential proteins that can interfere with or block bacterial chromosome replication through drug inhibition can be of great help in designing efficient drugs targeting a range of diseases caused by bacteria. In almost all bacterial species, chromosomal replication is triggered by the binding of the primary initiator protein (DnaA) to chromosomal replication origin (*oriC*), thus, DnaA and *oriC* are the main forces behind the formation of multimeric complexes required for the initiation of DNA replication [67]. The control of DnaA, which has multifunctional proteins required for chromosome replication, is the most prominent goal for inhibiting chromosome replication [68]. The four domains of DnaA have already been well summarized in literature, especially the N-terminal domain [69], [70]. To date, researchers have made considerable efforts to understand bacterial replication-related proteins, and the replication initiation of many bacterial species, including *Escherichia coli*, *Bacillus subtilis*, and *Caulobacter crescentus*, has been well studied [21], [68], [70], [71]. Meanwhile, the replication-related proteins are always present as a cluster aroud *oriC.* We have massively updated the information about the *oriC*s of bacteria in the online database DoriC 10.0 [72] based on the predicted results of Ori-Finder [73], [74], which can provide excellent opportunities to better explore the replication-related proteins of more bacteria.

### 2.4 Transcriptomics analysis

According to the genetic “central dogma”, transcription plays an important role in controlling the transmission of genetic information, which is the first key step in gene expression [75]. At present, transcriptomics has emerged as the leading and exciting topic in the life science field [76]. Transcriptomics is the study of cellular gene transcription and transcriptional regulation at the RNA level, and can provide a comprehensive and rapid understanding of the molecular mechanism of diseases and drug action at the transcriptome level [77]. Therefore, transcriptomics analysis has developed into a useful tool for acquiring novel antimicrobial targets [22], [78]. To better assist in the discovery of drug targets and drug design in different ways, a few technologies for transcriptomics studies, such as RNA-sequencing (RNA-seq) method for gene expression [79] and gene microarray or chip technology [80] have been developed and widely used to quickly search the transcriptomics. Practical applications of NGS-based RNA-seq method and microarray analysis in predicting genetic targets have been well reviewed [22], [75]. Recently, detailed target analyses of *Escherichia coli*, *Clostridium difficile*, *Mycobacterium tuberculosis*, *Mycobacterium smegmatis* and other pathogenic bacteria [81], [82], [83], [84], [85], [86], [87], [88] have been performed using bacterial transcriptomics, relevant techniques, and transcriptomics experiments. These successful cases of transcriptomics analyses again demonstrate that transcriptomics is a promising approach for predicting bacterial drug targets.

### 2.5 Riboswitches analysis

Riboswitches can mediate the expression of crucial and essential genes that are critical to the survival and virulence of bacterial pathogens [89], [90], and inhibiting the synthesis of bacterial ribosomal proteins can achieve antibacterial purposes [91]. Hence, bacterial riboswitches are considered as promising and capable antimicrobial targets for new drugs. In fact, riboswitches are widely found in the bacteria genomes and absent in human genome, which will reduce the probability of potentially harmful effects in humans and is one of the advantages of riboswitches as a useful tool for exploring bacterial drug targets [92]. In addition, riboswitches can bind to small molecules with high selectivity and are controlled by simple metabolism [93]. Given these advantages, the use of riboswitches as drug targets has attracted increasing attention, and some riboswitches-related work can provide valuable clues for future research [23], [94], [95]. It has been proven that few of the most widespread riboswitches, including lysine, cobalamin, SAM, and SAH, are useful antibacterial drug targets. In addition, certain methods for exploring potential riboswitches, such as Riboswitch Scanner [96] and drug design including high-throughput screening method have been well summarized [97]. Based on a powerful covariance model (CM), a comprehensive online database (RiboD) has recently been developed as a useful resource for predicting ribosomes in bacteria [98]. Using existing methods or developing new ones to dig deeper into riboswitches-related targets in bacteria will greatly help in the treatment of diseases associated with bacteria.

## 3 Target analysis

**Fig. 3:**
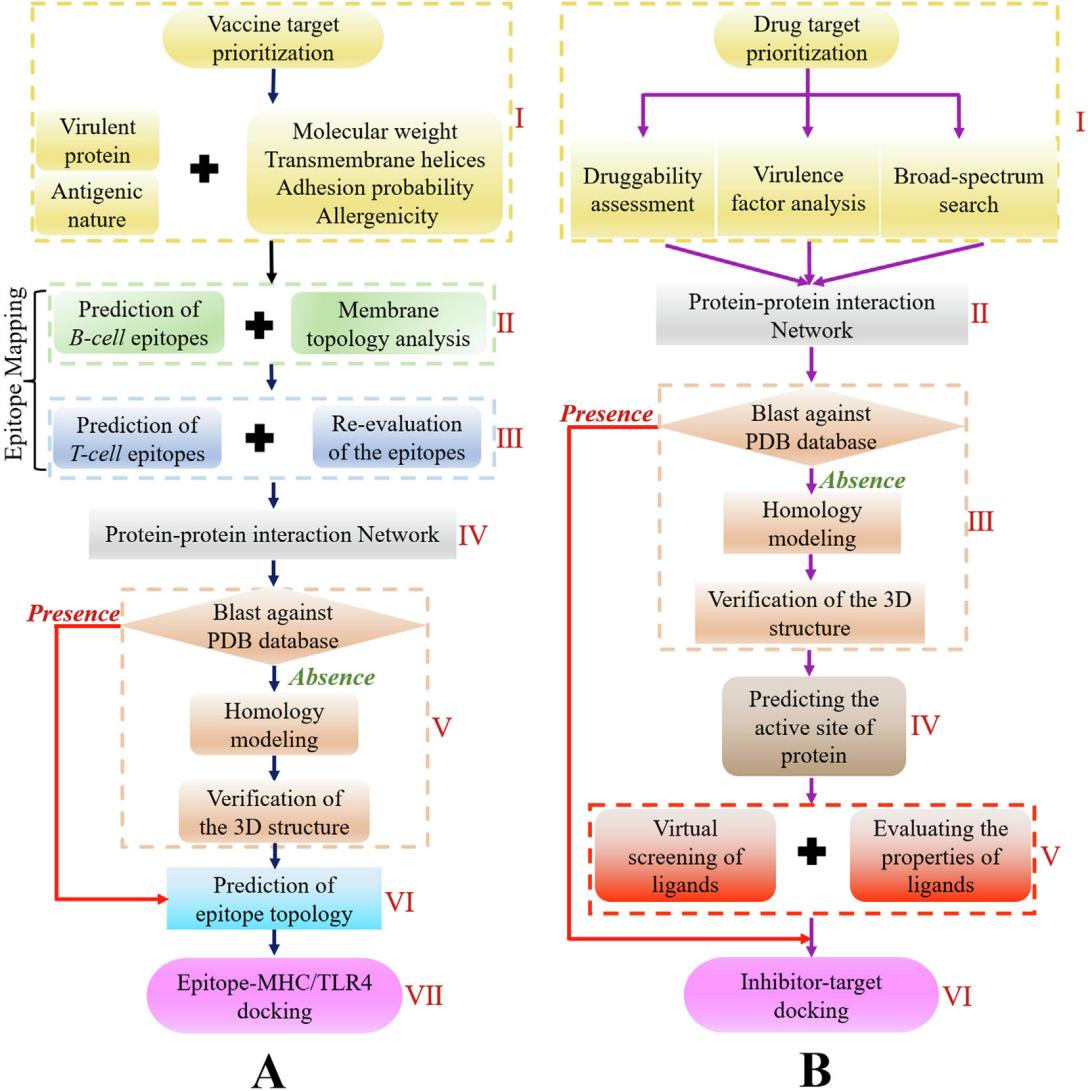
Target analysis flowchart for target optimization and construction of 3D epitopes structures, target proteins and drugs: (A) Analysis of vaccine target, (B) Analysis of drug target.

Once we have identified the vaccine and drug targets, the next important thing is to search for novel inhibitors and vaccines based on these possible targets. Notably, there are still considerable differences in the design of vaccines and drugs because of their unique properties, and the screening of vaccine targets is more complicated than that of drug targets. In this section, we present a detailed and systematic summary of the fundamental processes of target analysis, which are presented as flowcharts in Fig. 3.

### 3.1 Prediction of vaccine candidates

#### 3.1.1 Vaccine target prioritization

Virulence is an important factor in the study of pathogenesis. Compared with non-virulent proteins, virulent proteins are more likely to cause serious infections and promote the survival of pathogens in the host, making it an attractive target for vaccine design [99], [100]. Therefore, virulence analysis has been incorporated into the flowchart as a necessary step (Fig. 3A-I). Currently, a few free databases, such as the Virulence Factor Database (VFDB) of pathogenic bacteria [101] and the Microbial Virulence Database (MvirDB) [102] are available that can be used to gain information on the virulence of proteins. In addition to these two databases, Garg et al. also performed protein virulence prediction using the Virulentpred server [103] with a threshold of ≥1. These selected virulent proteins are then subjected to antigenicity evaluation using the online VaxiJen server [104], where proteins with antigenicity scores ≥0.4 are marked as potential antigens that can effectively stimulate the human immune system.

Meanwhile, the physiochemical properties of all potential targets, including molecular weight, transmembrane helices, adhesion probability and allergenicity, are analyzed to assist in experimental validation. These factors may improve the vaccine prediction accuracy and reduce any negative effects. For ensuring purification during the experiment, the focus in a majority of studies has been concentrated on only selecting proteins with molecular weight ≤ 110 kDa as effective drug targets [105], [106], and these shortlisted proteins measured by freely available ExPASy server [107] will simplify the purification and development process. Furthermore, the number of transmembrane helices in the proteins can affect the cloning and expression of the target, and their presence in large quantities may lead to the failure of experimental validation; thus, selecting proteins with fewer transmembrane helices is more feasible [108]. For this purpose, two popular servers, TMHMM [109] and HMMTOP [110] are widely used to evaluate the number of transmembrane helices [106], [111], [112]. In addition, it has been reported that the interaction between the bacterial surface proteins known as adhesions and the host receptors contributes greatly to the bacterial attachment, and the antibodies generated due to these adhesive proteins can prevent infections and diseases [113]. Therefore, the adhesion probability of the proteins should be taken into account, which can be effectively predicted using the data available on the Vaxign [114] or SPAAN server [115]. Finally, the allergenicity analysis of all filtered protein is performed by accessing the Allertop server [116], online AlgPred [117] or SORTALLER [118] to reduce the allergic reactions, and proteins that could cause allergic behavior are removed.

In this section, virulent and antigenic proteins with prospective physiochemical characteristics are scanned for subsequent analysis.

#### 3.1.2 Prediction of B- and T-cell epitopes

Since Barh et al. proposed the peptide vaccine design for *Neisseria gonorrhoeae* in 2010 [119], epitope-based vaccine design (EBVD) has emerged as the most popular and effective strategy in vaccine design [120], [121], [122]. Epitope vaccines have several advantages over traditional vaccines, such as atoxicity, safety, stability and easy production. They can also directly stimulate the host to create a specific immune response, thus confirming the suitability of EBVD for future development directions [119]. It is known that the antigen specificity and diversity are determined by B- and T-cell epitopes. Therefore, discovering the B- and T-cell epitopes capable of stimulating B- and T-cell immune responses is an imperative step in the development of such vaccines. In the following section, we summarize the prediction process of B- and T-cell epitopes in detail.

The proteins retained in the prioritization process are ideal vaccine candidates for the preparation of epitope-based vaccines, and these proteins are used to conduct an epitope analysis to predict the B-cell epitopes by employing the software BCPreds [123] or recent BepiPred-2.0 [124]. The selected B-cell epitopes with a BCPreds threshold score > 0.8 are then subjected to membrane topology analysis to determine their exposed topology by TMHMM (Fig. 3A-II).

The T-cell epitopes are then screened from B-cell epitopes with exposed surface based on the principles put forward by Barh et al. [119]. It has been affirmed that the binding affinity of reactive peptides to both classes of major histocompatibility complex I and II (MHC-I and II) molecules plays a vital role in immune response [125]. For the selection of an efficient T-cell epitope (Fig. 3A-III), the first step is to identify the binding epitope alleles to MHC-I and MHC-II by using the Propred1 [126] and Propred [127] servers, respectively. T-cell epitopes that can bind to more than fifteen MHC molecules simultaneously, especially to HLA-DRB1*0101, are cataloged. It is worth noting that DRB*0101 is the most frequent MHC-II allele, and an antigen can produce a more effective immune recognition and immune response when bound to DRB*0101 instead of other alleles. Next, calculation of the half-maximal inhibition concentration (IC_50_) for all probed T-cell epitopes is performed utilizing MHCPred [128] and the epitopes with an IC_50_ score < 100 nM are considered. Then, the virulence, antigenicity, adhesion probability, and allergenicity of the B-cell-derived T-cell epitopes are re-confirmed using VirulentPred, VaxiJe, Vaxign and Allertop servers, respectively. Meanwhile, ProtParam, Comprehensive Antibiotic Resistance Database (CARD) [129] and CLC Sequence Viewer are separately chosen to further estimate the chemical stability, resistance sequence, and conservation of the final selected epitopes.

Finally, ideal T-cell epitopes are successfully selected from a large number of vaccine targets.

#### 3.1.3 Interaction network

This work extends further to the selection of epitope proteins with strong cellular interactions (Fig. 3A-IV). Proteins with strong connections to neighboring proteins are regarded as hub proteins, which contribute greatly to the protein-protein interaction (PPI) network and have a direct relationship with the lethality of the pathogen [130]. If the activity of the hub proteins in the PPI network is inhibited, the entire network will be affected. Given the importance of key proteins, understanding the PPI network of the target candidates at the cellular level is also crucial, and has important implications for future vaccine and drug development [131]. The interaction analysis of all remaining epitope proteins can be achieved by searching a large number of protein relationships with the Search Tool for the Retrieval of Interacting Genes (STRING) [132], and the output results contain direct and indirect interactions from different sources. In the protein interaction network, proteins with the highest confidence score (0.9) are selected for further analysis [105], [112].

#### 3.1.4 Homology modelling and epitope topology analysis

With the aim of visualizing the topology of the predicted epitopes, the 3D structures of the epitope proteins need to be known (Fig. 3A-Ⅴ). As the initial step, a BLASTp search against Protein Data Bank (PDB) [133] is performed to seek structural information about the epitope proteins or suitable structural templates for epitope proteins that are unidentified to date, which is important for the prediction of immunogenic domains. For protein structures that are unavailable in the PDB library, the corresponding structures can be constructed by homology modeling. Online available servers, including I-TASSER [134], Phyre2 [135], Modweb [136], RaptorX [137], Modeler [138], M4T [139] and Swiss-Model [140] can help predict the 3D structure of the vaccine candidates. Subsequently, common web servers RAMPAGE, ProSA [141] verify 3D [142], ERRAT algorithm [143], WHAT_CHECK [144] and PROCHECK program [145] can be combined to accurately validate the 3D structure. Using the Ramachandran plot and Z-score analyses, the structure with the most residues mapped in favorable regions and a few residues in disallowed regions are selected as the best structure for each protein. In addition to the tools described above, PEPFOLD [146] can also be utilized to design the 3D structures of the epitopes according to amino acid sequence.

Once we know the 3D structure of the epitope proteins, this information can help us to calculate and predict the corresponding epitope topology (Fig. 3A-VI) [147]. To ensure the epitopes that effectively trigger the host immune system have exposed surfaces, the Pepitope server [147] is used to perform an exomembrane topology analysis on the shortlisted epitopes and their respective folded proteins.

#### 3.1.5 Molecular docking

A promising molecular docking method is subsequently performed to view the binding affinity and binding modes of epitopes to the MHC alleles [119] or Toll-like receptor 4 (TLR4) (Fig. 3A-VII) [105], [112], [148]. The precise epitope-protein docking can be achieved by ClusPro 2.0 [149], or a combination of PatchDock [150] and FireDock [151], or a combination of Autodock Vina [152] and GalaxyPepDock [153]. The detailed binding information of the peptide-protein complex can be viewed through UCSF Chimera [154] and LigPlot [155].

### 3.2 Prediction of drug candidates

#### 3.2.1 Drug target prioritization

As depicted in Fig. 3B-Ⅰ, the overall prioritization of predicted drug target is mainly considered from three factors: druggability, virulence factor (VF) and broad spectrum. The ideal drug target should integrate closely with drug-like molecules to make the drug more effective, and the binding affinity of the target proteins to the drug-like molecules can be reflected by druggability [156]. To find the proteins that can develop into potential drug targets, all putative proteins undergo the BLASTp similarity analysis against the bacterial drug targets in the DrugBank database [157] to assess the druggability of each protein, and predicted proteins with a high similarity to the bacterial drug targets are regarded as druggable targets for subsequent analysis.

Virulent proteins can regulate the infection pathway and play a decisive role in the survival of the pathogens in the host [99]. Thus, VF analysis has been proven as a promising approach for identifying therapeutic drug targets. To probe the virulence-related proteins, VFDB is applied for similarity comparison using the BLAST tool with a bit score > 100, and the output data will contain multiple types of virulence factor, such as adherence and protease.

Bacterial pathogens can generate different simultaneous infections in the host, thus, screening for broad-spectrum targets is now considered preferable. In this step, a broad-spectrum search of predicted proteins is conducted to investigate the potential broad-spectrum targets by BLASTp against bacterial pathogen proteomes with an e-value of 0.005 [14], [16].

After this progressive evaluation, the non-homologous and essential proteins that pass successfully through these filtration conditions and demonstrate unique metabolic pathways to the host are listed as prospective drug targets.

#### 3.2.2 Interaction network, homology modelling and 3D structure assessment

The PPI network analyses, homology modeling and 3D structural assessment of the drug candidates are similar to the corresponding analyses used for the prediction of vaccine targets (Fig. 3B-II and III).

#### 3.2.3 Predicting the binding site of target proteins

Once the final model of the predicted proteins is established, the next step is to predict the binding site of the proteins, which is essential for understanding the protein function (Fig. 3B-IV). Proteins contain a large number of residues, whereas the binding site is composed of those residues that can bind specifically to the drug. Therefore, understanding the interactions between the inhibitors and the proteins is crucial in drug design. Candidate interaction-based binding sites can be forecasted with the following programs: COACH [158], Computed Atlas of Surface Topography of proteins (CASTp) [159], Active Site Finder tool, DoGSiteScorer [160], fpocket [161], MetaPocket [162], and GHECOM [163].

#### 3.2.4 Virtual screening of ligands, evaluating the properties of ligands and molecular docking

Virtual screening (VS), also known as computer screening, is one of the latest advances in drug discovery (Fig. 3B-Ⅴ). Generally speaking, VS involves screening candidate ligands through ligand databases and investigating the possibility of these molecules binding or docking with the target proteins. ZINC is a broad platform for drug screening, and can accomplish a multi-method molecular search according to structure, properties, targets, etc. Initially, small molecules in the MOL2 format are downloaded from the ZINC database [145]. Then, the selected ligands are converted into the PDBQT format and undergo VS using the AutoDock Vina or AutoDock software tools [164], and these two software tools can realize the batch docking of molecules. The docking results are sorted in ascending order based on the binding free energy (ΔG_bind_) between the inhibitor and the receptor, and the first ten candidates are generally selected as the ideal inhibitors.

Molecular properties of the ligands are important for every step of the design, synthesis and clinical application of an effective drug. For the purpose of minimizing the negative effects of the selected ligands, the absorption, distribution, metabolism, excretion and toxicity (ADMET) characteristics that can influence the pharmacokinetics of the designed drugs are further evaluated by employing the SwissADME program [165] or the PreADMET server. The DrugBank database can also be used to assess the pharmaco-chemical properties of the drugs. Ultimately, the best predicted ligands with better pharmacokinetics and pharmaco-chemical features are acquired.

The docking between an inhibitor and a target protein is more complicated than the binding between two proteins, and their docking relationship is similar to that of a lock and key (Fig. 3B-VI). The inhibitor and the target protein should be paired complementarily, and the attachment of the inhibitor to the binding pocket should be as close as possible. Once the receptor proteins and inhibitors are ready, flexible molecular docking can be done through AutoDock, AutoDock Vina, GOLD [166] software, etc. Structures with the lowest binding free energy during molecular docking are considered the best and most stable initial structures for MD studies.

## 4 MD analysis

**Fig. 4:**
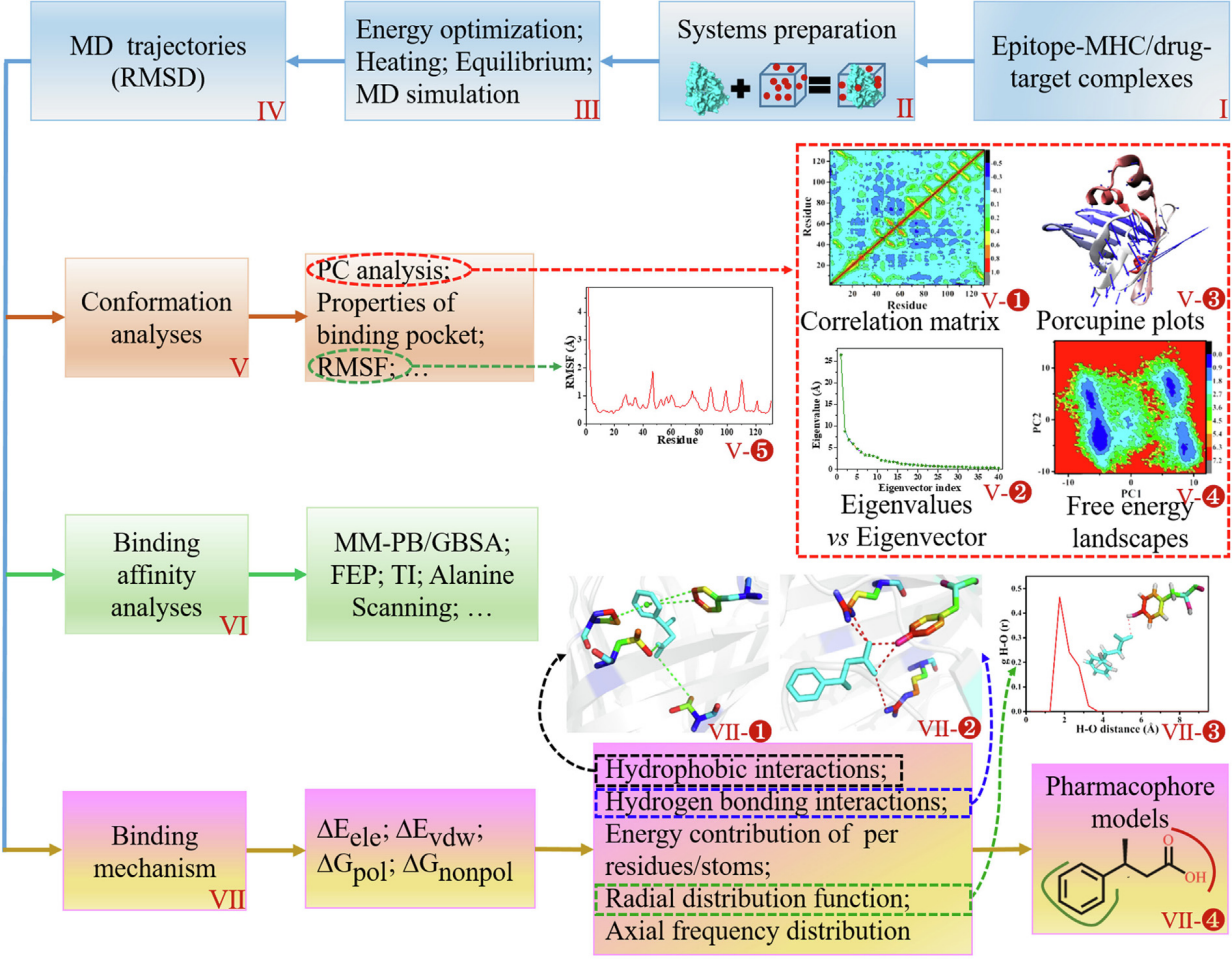
Flow diagram of MD analysis for studying the binding mechanism of inhibitors/epitopes to proteins and conformational changes in proteins.

MD analysis is a computer-based simulation method used widely in several fields, such as physics, chemistry, and biology. With the development of computer simulation technology, MD simulation [167], [168], [169], [170] has become a common tool for studying the binding mechanism of the inhibitors/peptides to the proteins and conformational changes of target proteins based on equilibrium MD trajectories. MD simulation can respond well to the dynamic characteristics of biomolecules, which helps provide a better theoretical basis for efficient vaccine and drug design. At present, existing mature software packages such as AMBER [171] and GROMACS [172] can provide strong technical support for MD simulations. To understand MD simulation better, we now summarize the basic MD simulation process and the advanced methods used for analyzing the conformation of target proteins and the binding affinity between the protein and the drug/vaccine (Fig. 4).

### 4.1 System preparation

Prior to the MD simulation, the selected systems should be properly prepared by applying the following four steps: adding missing hydrogen atoms to their respective heavy atoms; setting force fields for the proteins, inhibitors, or peptide epitopes; adding a certain amount of Na and Cl ions to neutralize the system; and immersing all the systems into a water box (Fig. 4II).

After the systems are well prepared, three critical operations (energy optimization, heating, equilibrium) are executed stepwise to ensure that the MD simulations are performed in an ideal experimental environment (Fig. 4III). First, the energy of all the studied models is optimized to eliminate any possible adverse effect on the structural deformation and simulation stability by combining the steepest descent and conjugate gradient methods. Subsequently, the system is gradually heated until the expected temperature of 300 K is reached. In the next step, the simulation is continued at the same temperature and characteristics including pressure, energy, and structure are evaluated. The simulation continues until these characteristics stop showing any changes over time. Finally, long-time MD simulation is performed at room temperature (300 K) and atmospheric pressure (1 atm). To better understand the universality of MD simulations and the various analytical methods based on the MD trajectories, we arbitrarily selected an example (PDB ID: 3P6F) to simulate the MD of 150 ns and the calculated results are presented in Fig. 4.

### 4.2 Root-mean-square deviation

The root mean square deviation (RMSD) value represents the deviation of backbone atoms in the proteins relative to their respective initial optimized structures, and is a commonly used method to evaluate the stability of the system. Smaller the RMSD value, the more stable the system is during the simulation. Generally, the equilibrium MD trajectories are selected for later analysis (Fig. 4IV).

### 4.3 Conformation analysis of proteins, and assessment of binding affinity and binding mechanism between inhibitors/peptides and proteins

To be effective, a drug must reach the binding site of the target protein and generate a strong interaction with the residues at the active site to form a stable complex. Meanwhile, proteins binding with the inhibitors/peptides will cause changes in their conformation. Therefore, a deeper understanding of the binding affinity and binding mechanism of inhibitors/peptides to the proteins and the conformation changes in the proteins induced by the binding will be of great help in the design of effective drugs and vaccines.

#### 4.3.1 Conformation analysis

The most popular tool for performing conformation analysis is the principal component analysis (PCA) [173]. Fundamentally, this method constructs a covariance matrix based on the coordinates of Cα atoms using a dimensional reduction method, which can then reflect the deviations of the Cα atoms from their respective average positions. Thus, cross-correlation matrices (Fig. 4Ⅴ-1) corresponding to the correlated motion between residues can be constructed. In a diagonalized covariance matrix, the eigenvalues and the eigenvector plot (Fig. 4Ⅴ-2) representing the motion intensity and direction of the residues, respectively, can be obtained, and then a porcupine plot (Fig. 4Ⅴ-3) can be established to characterize the movement of the residues. By projecting MD trajectories onto the first and second principal components (PC1 and PC2), the binding free energy landscapes (Fig. 4Ⅴ-4) of the proteins can also be constructed to better reflect their conformational distribution. Existing work has shown that a combination of RMSD and gyration radiuses (GR) can also be used to construct the free energy landscapes [174].

In addition to PCA, a few other methods are also used to analyze the protein structure based on the equilibrium MD trajectories. For example, the root mean square fluctuation (RMSF) of Cα atoms can be used to indicate the flexibility of the protein during the MD simulation (Fig. 4Ⅴ-5). The stability, continuity, correlation and volume of the binding pocket for each protein can also be separately evaluated by employing the D3Pockets server [175] and the POVME procedure [176] to characterize the structural changes in the proteins.

#### 4.3.2 Binding affinity analysis

The binding ability of inhibitors/peptides to proteins can be confirmed by calculating the ΔG_bind_ between the inhibitors/peptides and the proteins (Fig. 4VI). Numerous methods have been developed to predict the ΔG_bind_, including molecular mechanics Poisson Boltzmann/generalized Born surface area (MM-PB/GBSA) [177], thermodynamics integration (TI) [178], and free energy perturbation (FEP) [179]. Considering the computational resources and time, MM-PBSA and MM-GBSA have been the most widely used methods in recent years. In this method, the ΔG_bind_ between inhibitor and protein can be determined by the following formula.

(1) $$\Delta {\text{G}}_{\text{bind}}=\Delta {\text{E}}_{\text{ele}}+\Delta {\text{E}}_{\text{vdw}}+\Delta {\text{G}}_{\text{pol}}+\Delta {\text{G}}_{\text{nonpol}}-\text{T}\Delta \text{S}$$

The items on the right side of the equation represent the contributions of the electrostatic interaction (Δ${\text{E}}_{\text{ele}}$), van der Waals interaction (${\Delta \text{E}}_{\text{vdw}}$), polar interaction (${\Delta \text{G}}_{\text{pol}}$), nonpolar interaction (${\Delta \text{G}}_{\text{nonpol}}$) and entropy change (ΔS) to ${\Delta \text{G}}_{\text{bind}}$, respectively. Notably, the calculation of entropy is time-consuming; therefore, only 50–100 conformations are generally calculated by normal mode method [180]. In addition, the new interaction entropy (IE) method proposed by Duan et al. can also help in the calculation of entropy [181].

To further understand the influence of key residues on binding affinity, a computational alanine scanning method [182] based on MM-PBSA and MM-GBSA methods can also be applied to estimate the change in $\Delta {\text{G}}_{\text{bind}}$ and binding mechanism caused by the mutation of residues. Alanine mutant structures are generated by altering the coordinates of the wild-type (WT) residues, and the alanine residue parameters then replace all the parameters of the WT residue in the topology file. Subsequently, computational alanine scanning is performed based on the same snapshots as implemented in the MM-PBSA method. The difference in ΔG_bind_ can be determined by the following equation.

(2) $${\Delta \Delta \text{G}}_{\text{ala}}\text{=}{\Delta \Delta \text{G}}_{\text{bind}}^{\text{wt}}\text{-}{\Delta \Delta \text{G}}_{\text{bind}}^{\text{mut}}$$

where the first two terms (${\Delta \text{G}}_{\text{bind}}^{\text{wt}}$, ${\Delta \text{G}}_{\text{bind}}^{\text{mut}}$) are the binding free energies of WT and mutant complexes. The measurement unit for terms ${\Delta \text{G}}_{\text{bind}}$, ${\Delta \text{E}}_{\text{ele}}$, ${\Delta \text{E}}_{\text{vdw}}$, ${\Delta \text{G}}_{\text{pol}}$, ${\Delta \text{G}}_{\text{nonpol}}$, ${\Delta \Delta \text{G}}_{\text{ala}}$, ${\Delta \text{G}}_{\text{bind}}^{\text{wt}}$, and ${\Delta \text{G}}_{\text{bind}}^{\text{mut}}$ is kcal/mol.

#### 4.3.3 Binding mechanism analysis

Through continuous efforts of a large number of researchers, the binding mechanism between drugs/peptides and proteins has been extensively studied (Fig. 4VII). With the calculation of ΔG_bind_, analyses of ${\Delta \text{E}}_{\text{ele}}$, ${\Delta \text{E}}_{\text{vdw}}$, ${\Delta \text{G}}_{\text{pol}}$ and ${\Delta \text{G}}_{\text{nonpol}}$ interactions between inhibitors/peptides and proteins have been performed [168], [169], [183]. In these analyses, as the electrostatic and van der Waals interactions play a major role in the binding of drugs/peptides with proteins, further research on these two interactions has also been performed. Presently, the energy contributions of individual residues in proteins and individual atoms on residues to electrostatic and van der Waals interactions have also been calculated [184]. Simultaneously, detailed analyses of the hydrogen bonding (Fig. 4VII-1) and hydrophobic interactions (Fig. 4VII-2) between residues and inhibitors/peptides have also been performed to reveal the source of these two interactions. Furthermore, the radial distribution function (RDF) (Fig. 4VII-3) can partially contribute to the analysis and identification of hydrogen bonds [185]. Recently, a comprehensive method of axial frequency distribution (AFD) has also been proposed. This method can not only reflect the conformational characteristics, such as structural stability and flexibility, but also be used to analyze bi-molecular interactions including hydrogen bonds, van der Waals, and polar or ionic interactions [186]. We believe that due to long-term efforts, the prediction of binding affinity and binding mechanisms between inhibitors/peptides and proteins is no longer a puzzle.

After an in-depth analysis of the interaction mechanism, the optimal pharmacophore model [169], [187] is generated, as shown in Fig. 4VII-4. Generally speaking, the red-labeled region indicates that this region is easy to produce hydrogen bonding interactions with drug, while the green-labeled region make hydrophobic interactions with drug. Once the theoretical pharmacophore models of the relevant drugs are identified, pharmacophore-based VS can be performed to explore additional drugs, as proved by the great success of this method [188], [189].

## 5 Summary and outlook

Developing drugs or vaccines for highly contagious bacterial diseases in a short period of time can be challenging, and some drugs/vaccines can also show adverse effects during clinical treatment, which poses a great challenge to the clinical treatment, and necessitates a strict and careful monitoring of each step of the drug/vaccine design.

In this review, the methods for target discovery, target analysis, and MD analysis are summarized to present a complete and systematic scheme for the design of effective drugs and vaccines. In the first step, five common analytical methods, including comparative subtractive genome, core genome, replication-related proteins, transcriptomics, and riboswitches analyses are used to obtain promising drug and vaccine targets. Then, an in-depth analysis of selected targets is performed to minimize the negative effects of drugs and vaccines. Finally, each model is analyzed and verified by MD simulations to facilitate a deeper understanding of the binding mechanism of inhibitors/peptides to proteins and the structural changes in the proteins caused by the binding of inhibitors/peptides. We have also summarized the online software/database and corresponding websites used in each step to facilitate the readers to use and consult them, and the results are listed in Table 1.

**Table 1:** Online software and corresponding websites used in each step.

	Software/database	Website
Target Discovery	NCBI	https://www.ncbi.nlm.nih.gov
	UniProt	https://www.uniprot.org
	CD-HIT	http://cd-hit.org
	BLAST	https://blast.ncbi.nlm.nih.gov/Blast.cgi
	DEG	http://tubic.tju.edu.cn
	KAAS	http://www.genome.jp/kegg/kaas
	PSORTb	http://www.psort.org/psortb
	PGAP	http://pgapx.ybzhao.com
	EDGAR	http://edgar.computational.bio
	DoriC	http://tubic.tju.edu.cn/doric
	Ori-Finder	http://tubic.tju.edu.cn/Ori-Finder
	RiboD	http://ribod.iiserkol.ac.in
Target Analysis	VFDB	http://www.mgc.ac.cn/VFs/main.htm
	MvirDB	http://mvirdb.llnl.gov
	Virulentpred	https://www.bibsonomy.org
	VaxiJen	http://www.jenner.ac.uk/VaxiJen
	ExPASy	http://www.expasy.org
	TMHMM	http://www.cbs.dtu.dk/services/TMHMM
	HMMTOP	http://www.enzim.hu/hmmtop
	Vaxign	http://www.violinet.org/vaxign
	SPAAN	ftp://203.195.151.45
	Allertop	http://www.pharmfac.net/allertop
	AlgPred	http://www.imtech.res.in/raghava/algpred
	SORTALLER	http://sortaller.gzhmu.edu.cn
	BCPreds	http://ailab.ist.psu.edu/bcpred/predict.html
	BepiPred	http://www.cbs.dtu.dk/services/BepiPred
	Propred1	http://www.imtech.res.in/raghava/propred1
	Propred	http://www.imtech.res.in/raghava/propred
	MHCPred	http://www.jenner.ac.uk/MHCPred
	ProtParam	http://web.expasy.org/protparam
	CARD	https://card.mcmaster.ca
	CLC	https://www.clcbio.com
	STRING	http://string-db.org
	PDB	http://www.rcsb.org
	I-TASSER	http://zhang.bioinformatics.ku.edu/I-TASSER
	Phyre2	http://www.sbg.bio.ic.ac.uk/phyre2
	Modweb	http://salilab.org/modweb
	RaptorX	http://raptorx.uchicago.edu
	Modeller	https://salilab.org/modeller
	M4T	http://www.fiserlab.org/servers/m4t
	Swiss-Model	http://swissmodel.expasy.org
	RAMPAGE	http://mordred.bioc.cam.ac.uk/~rapper/rampage.php
	ProSA	https://prosa.services.came.sbg.ac.at
	Verify 3D	http://nihserver.mbi.ucla.edu/Verify_3D
	ERRAT	http://nihserver.mbi.ucla.edu/ERRAT
	WHAT_CHECK	https://swift.cmbi.umcn.nl/gv/whatcheck
	Pepitope	http://pepitope.tau.ac.il
	PEPFOLD	http://bioserv.rpbs.univ-paris-diderot.fr/PEP-FOLD
	ClusPro	https://cluspro.org
	PatchDock	http://bioinfo3d.cs.tau.ac.il
	FireDock	http://bioinfo3d.cs.tau.ac.il/FireDock
	Autodock Vina	http://vina.scripps.edu
	GalaxyPepDock	http://galaxy.seoklab.org/pepdock
	UCSF Chimera	http://www.cgl.ucsf.edu/chimera
	LigPlot	http://www.ebi.ac.uk/thornton-srv/software/LigPlus
	DrugBank	http://www.drugbank.ca
	COACH	http://zhanglab.ccmb.med.umich.edu/COACH
	CASTp	http://sts.bioe.uic.edu/castp
	ActiveSite Finder	http://www.scfbio-iitd.res.in/dock/ActiveSite.jsp
	DoGSiteScorer	http://dogsite.zbh.uni-hamburg.de
	fpocket	http://fpocket.sourceforge.net
	MetaPocket	http://projects.biotec.tudresden.de/metapocket
	GHECOM	http://strcomp.protein.osaka-u.ac.jp/ghecom
	ZINC	http://zinc.docking.org
	AutoDock	http://autodock.scripps.edu
	SwissADME	http://www.swissadme.ch
	PreADMET	https://preadmet.bmdrc.org
Molecular Dynamics Analysis	D3Pockets	http://www.d3pharma.com/D3Pocket/index.php

The development and application of effective drugs still need to undergo long-term and numerous clinical trials, and researchers have performed numerous clinical investigations on vaccines and drugs [190], [191], [192].We expect that this review will provide useful ideas and guidance for the clinical development of effective drugs or vaccines to cure potential infectious diseases or epidemics caused by bacteria.

## CRediT authorship contribution statement

**Fangfang Yan:** Conceptualization, Methodology, Investigation, Writing - original draft. **Feng Gao:** Supervision, Project administration, Funding acquisition, Writing - review & editing.

## Declaration of Competing Interest

The authors declare that they have no known competing financial interests or personal relationships that could have appeared to influence the work reported in this paper.
